# The effect of diabetes in the multifaceted relationship between education and cognitive function

**DOI:** 10.1186/s12889-024-20156-x

**Published:** 2024-09-27

**Authors:** Constantin Reinke

**Affiliations:** Institute for Sociology and Demography, University of Rostock, Ulmenstr. 69, 18057 Rostock, Germany

**Keywords:** Cognitive function, Education, Diabetes, Mediation, Population study

## Abstract

**Background:**

Education has been shown to be positively associated with cognitive performance. However, the pathways via lifestyle-related disease through which education is related to cognitive performance have not been sufficiently explored. Diabetes is an important lifestyle-related disease with increasing prevalence worldwide. Low education is associated with an increased risk of developing diabetes, while diabetes may also lead to a deterioration in cognitive performance. This study aims to explore if the associations between education and cognitive function is mediated by the diabetes status among older adults.

**Methods:**

The data utilized in this study were derived from the first two waves of the Dutch Lifelines Cohort Study (2006–2015). The analyzed sample included 26,131 individuals aged 50 years or above at baseline. The baseline assessment included measurements of educational attainment (exposure) and the potential mediator diabetes. The outcome of cognitive function was assessed using age-standardized reaction times from the psychomotor function and attention tasks, as measured by the Cogstate Brief Battery. The Cogstate Brief Battery was only conducted at the follow-up assessment, not at the baseline assessment. Faster reaction times correspond to higher cognitive performance. The study employed linear and logistic regression models, in addition to a causal mediation approach which estimated the average causal mediation effect (ACME).

**Results:**

Higher education was associated with a lower risk of diabetes (b= -0.1976, 95%CI= -0.3354; -0.0597) compared to low or middle education as well as with faster reaction times (b= -0.2023, 95%CI= -0.2246; -0.1798), implying better cognitive function. Diabetes was associated with slower reaction times (b = 0.0617, 95%CI = 0.0162; 0.1072). Most importantly, the mediation approach identified a significant indirect effect of education on cognitive function via the diabetes status (ACME= -0.00061, 95%CI= -0.00142; -0.00011).

**Discussion:**

The findings emphasize the potentially importance of diabetes in explaining the role of education in promoting healthy cognitive function and mitigating the risk of cognitive decline. Early detection and treatment of diabetes may be particularly beneficial for individuals with low or middle levels of education in order to maintain good levels of cognitive function.

**Supplementary Information:**

The online version contains supplementary material available at 10.1186/s12889-024-20156-x.

## Introduction

The doubling of the population aged 60 years and older by 2050 [1], in conjunction with the continued aging of the global population, will result in a significant increase in the global challenge of cognitive decline. The decline in cognitive function affects the individual’s daily activities, resulting in a diminished quality of life and loss of independence with a high burden on caregivers and health care systems. While brain changes associated with cognitive decline are part of normal brain aging [2], certain diseases such as diabetes can accelerate neurodegeneration and also be a driver of cognitive decline [3]. In 2021, it is estimated that there are 537 million people with diabetes, with a predicted increase to 783 million by 2045 [4]. Conversely, factors such as education can compensate for or delay cognitive decline [5, 6]. In many parts of the world, an impressive expansion of education has taken place during the last decades [7], which, however, leaves those with less education at a particular risk of disease and poor cognition [6, 8].

As cognitive aging and brain-altering processes are irreversible, the best strategy for reducing the risk of cognitive decline or delaying the onset or progression to clinical manifestations such as dementia is to identify and address those risk factors that are amenable to modification. The Lancet Commission identified less education as well as diabetes as important modifiable risk factors for dementia [9]. Thus, a better understanding of the pathways and possible links of these two factors with cognitive function may contribute to potential strategies for preventing or rather delay cognitive decline and dementia.

Diabetes is associated with deficits in cognitive function [10, 11] and a higher risk of cognitive impairment [12] as well as dementia [13]. Furthermore, there is evidence for a link between diabetes and brain atrophy which leads to deficits in cognitive function [14]. High blood glucose levels and hyperglycemic events affect the brain by cerebral microvascular dysfunctions [15] and can lead to brain atrophy [16]. On the other hand, diabetes is associated with higher risks of a series of cardiovascular diseases [17], which are known to be the main drivers of cognitive impairment and vascular dementia [18]. However, cognitive function can also be affected by high blood glucose levels and hyperglycemic events due to more complex pathways including oxidative stress and neuroinflammation [19]. Prior meta-analyses have indicated that diabetes exerts disparate effects on various domains of cognitive function, notably affecting psychomotoric function and attention [10, 11].

The association between education and cognitive function is well researched [20] and established by the concept of Cognitive Reserve [21]. The accumulation of education and experience of occupational complexity over a lifetime strengthens resilience to the pathology of cognitive decline due to age-related brain changes and delays the symptoms of cognitive decline or the clinical manifestation of dementia. In addition, there is evidence that education may also interact with the level of tau protein accumulation in the brain and its role in cognitive function [22]. Education has been identified as the most important proxy measure for cognitive reserve [23] and this is true for both individuals with and without diabetes as shown by the similar relationship between cognitive reserve level and a variety of executive cognitive function scores independent from the diabetes status [23].

The risk of diabetes is lower in individuals with higher levels of education [24, 25]. Although other determinants of socioeconomic status have been studied and associated with the diabetes risk, education has been the most frequently and consistently associated indicator [26]. The underlying mechanism for the relationship between education and diabetes is not fully understood, but factors related to lifestyle and healthy behaviors appear to play a crucial role. In particular, BMI has been identified as an important factor [27, 28].

It is reasonable to conclude that the most influential factor in the association of education and cognition is the direct link, as evidenced by the aforementioned connections of intellectual stimulation, such as the theory of cognitive reserve [20, 21]. Nevertheless, the pathways connecting education and cognitive functions are multifaceted and remain incompletely understood. Lower education is linked to a higher risk of lifestyle-related diseases [29], which are also associated with cognitive decline. This suggests that individuals with lower education levels may be more vulnerable to cognitive decline due to these conditions. Beside diabetes, also other life-style related diseases like cardiovascular diseases [30], vascular diseases [31], the number of chronic diseases [32] or obesity [33] are associated with worse cognitive function and represent possible determinates to play a role in the link between education and cognitive function. Among these diseases, diabetes is of particular interest because it is a modifiable risk factor for cognition in multiple ways. Prevention of diabetes and good glucose management in people with diabetes are both important to reduce the risk of cognitive decline [34, 35].

However, the interplay of education and diabetes and the consequence for cognitive function is still less researched and remain largely unknown.

Kowall & Rathmann examined the combined effects of education and diabetes on cognitive performance using longitudinal data from more than 27 countries from the SHARE project [36]. The authors found that people with diabetes had worse cognitive performance than people without diabetes, and that people with diabetes had even worse cognitive performance if they had lower levels of education. However, because the authors found no interaction effect between education and diabetes, they concluded that the effects were additive. A small retrospective case-control study including 1537 individuals from Japan examined the pathway between socioeconomic status and dementia by evaluating lifestyle-related disease as potential mediators [37]. However, the authors did not find a significant association between educational attainment and the risk of diabetes in their data, so a conclusion about the role of diabetes as a potential mediator is limited.

Because the pathways through which education is related to cognitive performance have not been well studied, this study addressed the question of whether some of the association between education and cognitive function may operate through the diabetes status. The hypothesis is that the diabetes status partly mediates the association between education and cognitive function.

**Fig. 1:**
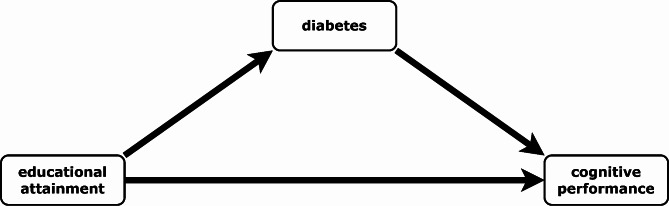
Hypothesized relationship between outcome (cognitive performance), mediator (diabetes) and exposure (educational attainment), source: Own illustration

## Materials and methods

### Data

Analyses were conducted using data from the Dutch Lifelines Cohort Study. *Lifelines is a multi-disciplinary prospective population-based cohort study examining in a unique three-generation design the health and health-related behaviours of 167,729 persons living in the North of the Netherlands. It employs a broad range of investigative procedures in assessing the biomedical*,* socio-demographic*,* behavioural*,* physical and psychological factors which contribute to the health and disease of the general population*,* with a special focus on multi-morbidity and complex genetics* [38]. The large dataset includes information on physical examinations, biological samples, cognitive tests and a comprehensive questionnaire. Data collection was conducted between 2006 and 2013 for the baseline assessments and between 2014 and 2015 for the second assessment. Lifelines was conducted in accordance with the guidelines of the Declaration of Helsinki and has been approved by Medical ethical committee of the University Medical Center Groningen (The Netherlands) under number 2007/152. All participants signed an informed consent form.

### Study design & sample

**Fig. 2:**
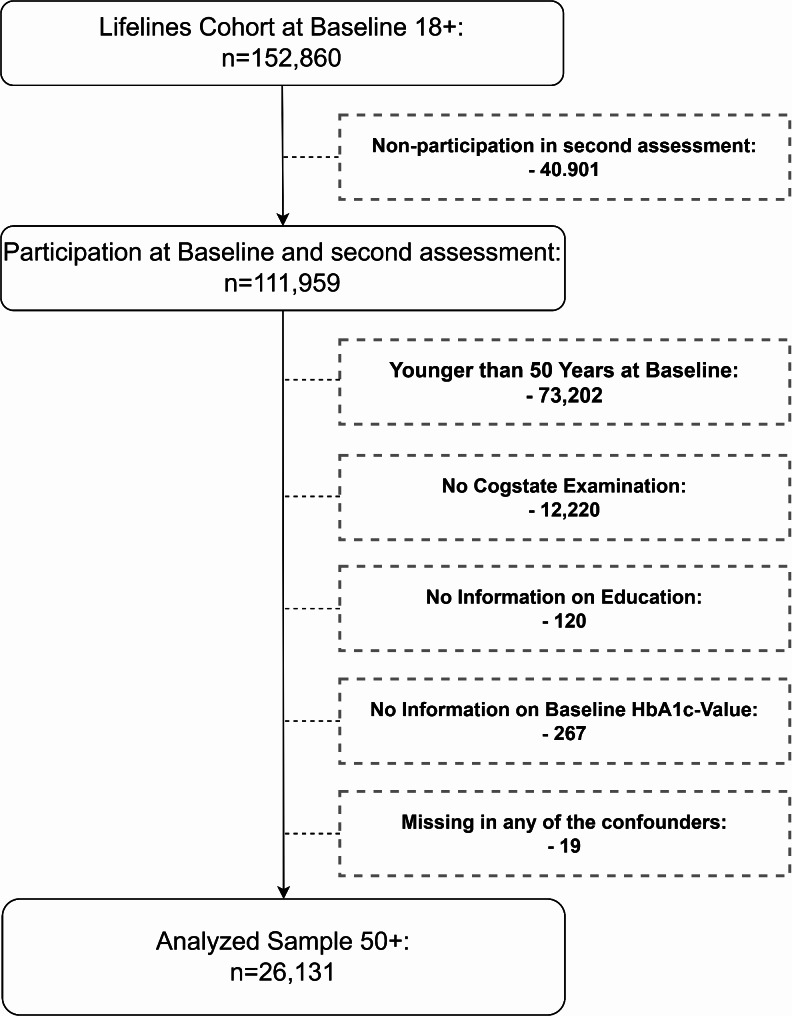
Selection of the study cohort, source: lifelines data 2006–2015, own calculation

The Lifelines cohort includes 152,860 individuals aged 18 years or older at baseline. Of these, 111,959 participated in the second assessment. All individuals younger than 50 years at baseline were excluded, as were individuals with missing data on outcome (no Cogstate examination), exposure (education), mediator (diabetes), or confounders. See Fig. 2. The final sample consisted of 26,131 individuals. To set up a study design with a causal time order, the variables for the exposure, the mediator and confounders were built by information from the baseline assessment. As the analysis only included individuals aged 50 or older at baseline, it can be assumed that their highest educational attainment was achieved well before the mediators and confounders were measured. Since the Cogstate examination was not conducted at baseline, the outcome measure was taken from the second assessment only. As a result, it was not possible to investigate the change in cognitive function over time or to adjust for baseline cognitive function.

### Data availability

Data may be obtained from a third party and is not publicly available. Researchers may apply to use the Lifelines data used in this study. For information on how to request Lifelines data and terms of use are available on their website at (https://www.lifelines.nl/researcher/how-to-apply).

### Cognitive function measure (outcome)

Individuals cognitive function was measured by tasks from the Cogstate Brief Battery at the second assessment only. The Cogstate Brief Battery is a validated computer based cognitive assessment [39]. It has been used in previous studies to detect mild cognitive impairment and cognitive impairment in Alzheimer’s disease [40] and also in the context of the Lifelines cohort [41]. The battery includes four different tasks to measure the cognitive domains of psychomotoric function (detection test), attention (identification test), visual learning (one card learning test), and working memory (one back test). Outcomes of each task were reaction time and accuracy.

Previous studies point out, that the associations of diabetes and cognitive function varied between the domains of cognitive function [10, 11]. There is evidence that primarily the domains of psychomotoric function and attention were affected by diabetes. Therefore, a composite score from these two domains was built by the detection and identification tasks of the Cogstate Brief Battery, similar to Maruff and colleagues [40]. In order to achieve this, the log-10-transformed (closer to a normal distribution) reaction time (milliseconds) of both tests were z-standardized in 5-year age groups (from age group 50 upwards) and then summed up (Distribution: Supplementary Figure S1). Accordingly, a positive value indicates a higher (slower) reaction time compared to the respective age group, representing poorer cognitive function. Conversely, a negative value displays a lower (faster) reaction time, implying better cognitive function.

### Educational attainment (exposure)

Individuals educational attainment was defined by the highest obtained degree at baseline. The information was self-reported through a questionnaire with the following possible responses: no education (1); primary education (2); lower or preparatory secondary vocational education (3); junior general secondary education (4); secondary vocational education or work-based learning pathway (5); senior general secondary education, pre-university secondary education (6); higher vocational education (7); university education (8); other (9). We categorized the education into two categories low-middle (1–6) and high education (7–8). Individuals who reported “other” were assigned to one of the two categories in a further step (see: https://wiki.lifelines.nl/doku.php?id=educational_attainment).

### Diabetes status (mediator)

Diabetes was defined as the presence of at least one of the following conditions at baseline: Self-reported diagnosis of diabetes, baseline HbA1c ≥ 6.5%, fasting plasma glucose ≥ 7 mmol/L, random plasma glucose ≥ 11.1 mmol/L or use of any medical diabetes treatment.

### Control variables

The statistical models were adjusted for age, sex, physical activity, obesity, smoking history, income and a set of comorbidities: depression, hypertension, stroke, heart failure and high cholesterol. The models on cognitive function were also controlled for the accuracy (number correct responses/number total response) of the tasks from the Cogstate Brief Battery. All confounders were measured at the baseline assessment.

Physical activity was defined as any amount of vigorous physical activity per week. So, the variable is classified into physical active vs. not physical active persons.

Obesity was defined by a body mass index of 30 or higher.

The smoking history was classified by using the cumulative risk measure of packyears, where one pack year implies smoking 20 cigarettes (or an equivalent number of other smoking derivatives) per day for one year. The variable was categorized into: Never smokers, persons with equal or less pack years than the median of the ever smokers, persons with more pack years than the median of the ever smokers, no answer/pack years not calculable.

Income was measured by the following question: “what is your net income per month? (if you share a household, include the net income of your partner(s))”. The variable is classified into the following categories: Lower than 1500€, 1500€ − 2500€, Over 2500€, Don’t know/no answer.

All comorbidities were measured by self-report (had the person ever had the condition).

### Statistical analysis

To explore the associations between education, diabetes status and cognitive function linear and logistic regression models were used. A causal mediation approach [42] was used to test whether there was an indirect effect of education (**E**xposure) on cognitive function (**O**utcome) through diabetes status (**M**ediator) controlled for the **c**onfounders. This approach which based on the idea or structural equation modeling estimates the average causal mediation effect (ACME) or indirect effect. The approach included several steps: First, two statistical models were fitted separately, one to model the mediator (1) and one to model the outcome (2). In a second step, model parameters for outcome and mediator were simulated from their sampling distributions. Third, potential values for the mediator were simulated prior to the potential outcomes, given these simulated mediator values, in order to then calculate the causal mediation effects from the simulated values. Finally, the point estimation for the ACME (and direct effect) as well as the confidence interval was calculated from the simulated distribution. The approach is described in detail by Imai et al. [42]

1 $$M = {\beta _0} + {\beta _1}E + \sum\limits_{i = 1}^n {{\beta _{1 + i}}} {c_i} + {\varepsilon _M}$$

2 $$O = {\alpha _0} + {\alpha _1}E + {\alpha _2}M + \sum\limits_{i = 1}^n {{\alpha _{1 + i}}} {c_i} + {\varepsilon _o}$$

In contrast to the classical framework of structural equation modeling, here the two models do not have to be linear regression models. Since the mediator (diabetes status) is a binary variable, it was estimated using logistic regression. While the outcome is a continuous variable, it was estimated by linear regression. Both models were estimated with robust standard errors. The causal mediation approach was performed using the mediation package in R [43].

To check the robustness of the results, a number of sensitivity analyses were applied. First, to test the sequential ignorability assumption that there are no unmeasured confounders of the mediator-outcome relation [44], the “medsens” function from the mediation package in R [43] was used. The function estimates a parameter which indicates the correlation of $\:{\varepsilon _M}$ and $\:{\varepsilon _o}$ at which the ACME would be zero. The function required a probit regression model instead of a logistic one.

As a further sensitivity analysis, a classical structural equation modeling approach with robust standard errors was used. However, in this classical framework both models have to be linear regression models, the mediator was the (continuous) HbA1c-level instead of the diabetes status otherwise the equations were identical to (1) and (2). After the simultaneously estimation of the outcome and the mediator model, the effect of education was decomposed in a direct and indirect effect and the Sobel test was used to check significance [45].

To test the robustness of the study design, the mediation analysis described above was also tested using indicators other than diabetes as potential mediators. In the same way as for diabetes, the indirect effect of education was tested via high blood pressure, high cholesterol and obesity.

In a further sensitivity analysis, the diabetes status was defined as the presence of at least one of the conditions mentioned in Sect. 2.5 at baseline or follow-up.

All analyses were performed using R 4.2.2 and Stata 17.

## Results

### Study cohort

Our analyzed sample included 26,131 individuals of which 18,486 had a low or middle educational attainment and 7645 had a high educational attainment, and there were 1449 diabetes cases. The mean age-standardized reaction time of the outcome measure was − 0.0003 (SD = 0.8796). The mean age at baseline was 58.1 (SD = 6.6) years, with a range of 50 to 88 years. The majority of participants were between 50 and 60 years of age. The mean age was 57.9 (SD = 6.5) without and 61.1 (SD = 6.9) for persons with diabetes. The individuals are divided into 14,503 women and 11,628 men. Table 1 shows characteristics of the study cohort. When looking at the outcome, it can be noticed that the mean age-standardized reaction time for high educated people (-0.2175) is clearly lower (faster/better) than for people with low or middle education (0.0895). So, in the cognition tests individuals with high education performed faster/better compared to their age-group while low-middle educated individuals performed slower/worse. A reverse pattern was observed in diabetes. Non-diabetic individuals demonstrated faster/better cognitive performance relative to their age group than diabetic individuals.

**Table 1:** Study cohort characteristics

Variable	Persons at baseline	Persons with diabetes at baseline (%)	psychomotor function and attention tasks* (SD)
**Age group**			
50–54	9569	285 (2.98)	0.0097 (0.8923)
55–59	6259	312 (4.98)	-0.0061 (0.8812)
60–64	5627	384 (6.82)	-0.0056 (0.8800)
65–69	3201	304 (9.50)	-0.0047 (0.8512)
70–74	1123	120 (10.69)	-0.0130 (0.8525)
75–79	287	34 (11.85)	-0.0034 (0.8384)
80+	65	9 (13.85)	-0.0064 (0.8466)
**Educational attainment**			
low-middle	18,486	1130 (6.12)	0.0895 (0.9109)
High	7645	318 (4.16)	-0.2175 (0.7559)
**Diabetes**			
No	24,682		-0.0070 (0.8769)
yes	1449		0.1137 (0.8715)
**Sex**			
Female	14,503	693 (4.78)	0.0325 (0.8790)
male	11,628	756 (6.50)	-0.0410 (0.8787)
**Physical activity**			
not active	17,930	1160 (6.47)	0.0445 (0.8907)
Active	8201	289 (3.52)	-0.0983 (0.8468)
**Obesity**			
No	21,920	839 (3.83)	-0.0098 (0.8775)
yes	4211	610 (14.49)	0.0495 (0.8890)
**Smoking history**			
never smoker	8842	415 (4.69)	0.0196 (0.8906)
equal or less pack years than the median of ever smokers	8020	337 (4.20)	-0.0118 (0.8781)
more pack years than the median of ever smokers	7929	587 (7.40)	-0.0343 (0.8595)
no answer/pack years not calculable	1340	110 (8.21)	0.1377 (0.9170)
**Household income per month**			
lower than 1500€ per month	2589	185 (7.15)	0.1221 (0.9149)
1500€ − 2500€ per month	7279	463 (6.36)	0.0552 (0.8811)
over 2500€ per month	11,670	503 (4.31)	-0.1123 (0.8342)
don’t know/no answer	4593	298 (6.49)	0.1274 (0.9331)
**Depression**			
No	23,618	1280 (5.42)	-0.0037 (0.8783)
yes	2513	169 (6.73)	0.0316 (0.8913)
**Stroke**			
No	25,836	1413 (5.47)	-0.0022 (0.8790)
yes	295	36 (12.2)	0.1621 (0.9246)
**Hypertension**			
No	17,641	626 (3.55)	-0.0102 (0.8791)
yes	8490	823 (9.69)	0.0203 (0.8804)
**Heart failure**			
No	25,816	1401 (5.43)	-0.0014 (0.8785)
yes	315	48 (15.24)	0.0888 (0.9601)
**High cholesterol**			
No	19,965	690 (3.46)	-0.0078 (0.8764)
yes	6,166	759 (12.31)	0.0240 (0.8896)
**Total**	**26,131**	**1449 (5.55)**	**-0.0003 (0.8796)**

*Source: lifelines data 2006–2015*,* own calculation*

* Mean age-standardized reaction time [log10-transformed milliseconds]

### Mediation analysis

Table 2 shows the estimated regression coefficients for the mediator model 1 (logistic) and the outcome model 2 (Ordinary Least Squares) with the corresponding confidence intervals (CI) as well as the direct and the indirect effect (ACME) of education on cognitive function. From model 1 it can be derived that higher education is associated with a lower risk of diabetes compared to low-middle education, indicated by the negative regression coefficient (-0.1976, *p* = 0.005). The regression coefficient (-0.2023, *p* < 0.001) in model 2 shows a significant association between education and cognitive function, implying that those with higher education have a lower reaction time to the outcome measure, and therefore better cognitive function than those with low or middle education. The regression coefficient for diabetes in model 2 is 0.0617 (*p* = 0.008). This demonstrates that individuals with diabetes have a significantly higher reaction time to the outcome measure and therefore worse cognitive function than individuals without diabetes. The average mediation effect of education trough diabetes was − 0.00061 and the direct effect of education was − 0.20247. This results in a total effect of -0.20307. In particular, the coefficient indicates that individuals with higher levels of education, in comparison to those with low-to-middle levels of education, completed the examined tasks, on average, 0.20307 standard deviation faster (better cognitive function) than the average in their respective age group. All these effects were significantly different from zero. However, the ratio of the indirect effect to the total effect implies that the indirect effect contributes less than 1% to the total effect. The average mediation effect (-0.00061) represents the difference in the effect of education on cognitive function through the mediator. In other words, it is the total effect minus the direct effect.

The first sensitivity analysis tested the sequential ignorability assumption of the causal mediation approach. The estimated parameter ρ that would lead to an ACME of zero was 0.036 (Supplementary Figure S2). So even a weak pre-treatment confounder could render the effect insignificant.

In a second sensitivity analysis, a classical structural equation modelling approach was applied with HbA1c level instead of diabetes status as a potential mediator. The results of the Sobel test showed that HbA1c was also a significant mediator for education (Supplementary Table S1). The proportion of the indirect effect from the total effect was also less than 1%.

A further sensitivity analysis was conducted to test the robustness of the study design by using other lifestyle-related diseases as potential mediators. The results showed that there were no indirect effects (ACME not significantly different from zero) of education on cognitive function via hypertension, high cholesterol, or obesity (Supplementary Table S2 – Table S4).

The sensitivity analysis, which defined diabetes status based on baseline and follow-up information, resulted in a larger number of cases of diabetes (2013 vs. 1449). However, the results of the mediation analysis did not differ significantly from those presented in Table 2 (see Supplementary Table S5).

**Table 2:** Results of regression models for the mediator and the outcome variable

	model 1 * (mediator)	model 2 † (outcome)
*dependent variable*:	diabetes status	cognitive function
*model type*:	logistic	OLS
	**Reg. coef. (95% CI)**	**Reg coef. (95% CI)**
**High education** (Ref.: low-middle)	-0.1976 (-0.3354; -0.0597)	-0.2023 (-0.2246; -0.1798)
**Diabetes** (Ref.: No-Diabetes)	-	0.0617 ( 0.0162; 0.1072)
**(Pseudo) R-squared**	0.1108	0.0847
**Number of observations**	26,131	26,131
**ACME of education** **(indirect effect)**	-0.00061 (-0.00142; -0.00011) 0.3% of the total effect	
**direct effect of education**	-0.20247 (-0.22516; -0.18052) 99,7% of the total effect	
**total effect of education**	-0.20307 (-0.226145; -0.18077)	

*regression coefficients and 95% confidents intervals & direct*,* indirect and total effect of education from the causal mediation analysis*,* source: lifelines data 2006–2015*,* own calculation.*

* Model controlled for: Age,* sex*,* physical activity*,* obesity*,* smoking history*,* income*,* and hypertension*.

*† Model controlled for: age*,* sex*,* physical activity*,* obesity*,* smoking history*,* income*,* comorbidities*,* and cognition test accuracy*.

## Discussion

The question of this study was whether diabetes partly mediates the link between educational attainment and cognitive function in individuals aged 50 years and older, using a large data set from the Netherlands. The results revealed significant positive effects of higher education on cognitive function as well as a lower risk of diabetes for higher educated individuals. The most noteworthy finding was the identification of a significant indirect effect of education on cognitive function via diabetes, although this effect was relatively small.

Although the mediating effect of diabetes on the relationship between education and cognition has not been considerably studied, an earlier study did examine the interaction effect between education and diabetes [36]. They did not find one and therefore concluded that the effects of the two risk factors were purely additive. This conclusion does not rule out that the connection may be partly a mediated association. In addition to the increased risk of developing diabetes among individuals with lower levels of education, potential reasons for the association to cognition may lie in the disparities in health literacy and adherence to diabetes therapy between the educational groups. Supporting Kowall and Rathmann, worse glycemic control which is more prevalent in lower educated people with diabetes [46, 47] and the association of worse glycemic control with cognitive dysfunction [48] may be a mechanism here. Treatment recommendations for glycemic control are challenging and include diabetes self-management by monitoring of blood glucose, use of medication as well as physical activity and nutrition/diet [49]. This health-related behaviors are linked to education [46, 50, 51]. Furthermore, the compliance of diabetes self-management decreases over time [52] and there is evidence that individuals with lower levels of education are at an increased risk of developing diabetes complications [53] which point out the possible link to cognitive function. This may give a higher potential for reducing the burden of cognitive decline in lower educated people by avoiding diabetes as well as diabetes complications through improving diabetes self-management and adherence.

Nakahori and colleagues [37] concluded that diabetes plays a minimal role in the link between educational attainment and dementia. This was not surprising as their study showed no significant association between education and diabetes, or between education and other dementia risk factors such as smoking. However, the authors emphasized that their findings are limited to Japan. In contrast to the findings of Nakahori et al., this study confirmed the existing evidence for the connection of education and diabetes [24, 25, 31] and argued that diabetes plays a significant role in the link between education and cognitive function. It should be noted that Nakahori et al. employed dementia as outcome, rather than cognitive function. Nevertheless, both are closely related and share a significant number of risk factors. A further reason for the disparate findings may be attributed to the differing definitions of diabetes applied. While Nakahori et al. only include diagnosed cases of diabetes, this study also incorporates individual’s laboratory results, which cover undiagnosed cases of diabetes. The link between undiagnosed diabetes and cognitive function appears to be particularly strong [54].

One of the main strengths of our study is its large sample size. The lifelines cohort covers about 10% of the population of the northern Netherlands. In this study more than 26,000 people were included and analyzed using information from questionnaires, measurements, and blood sample data. Central to the validity of the study is the utilization of a validated and well-established measure for assessing cognitive function. A composite score of cognitive function was constructed using tests from the Cogstate Brief Battery. This score was derived from two domains: psychomotor function and attention. There is a body of evidence indicating that these domains are associated with diabetes [10, 11]. An additional strength of our study is the comprehensive definition of diabetes, which includes both diagnosed and undiagnosed cases by incorporating the HbA1c-level from the blood sample. Furthermore, the statistical models were adjusted for a set of life style-related confounders. The sensitivity analyses concerning a further statistical approach, as well as the robustness of the study design using other possible mediators or extended definition of diabetes represent further strengths of this study.

Despite the strengths of this study, it is important to acknowledge its limitations. The Cogstate Brief Battery was not assessed at baseline; thus, it was not possible to examine changes in cognitive function in the context of the analyses. Moreover, the cognitive function at follow-up is supposed to be affected by the cognitive function at baseline; however, it was not possible to adjust the analysis for this information. In light of the findings of this study, it can be concluded that the outcome measure of cognitive function encompasses both the baseline information and the change from baseline to follow-up. Consequently, it is not possible to interpret these elements separately. It is relevant to consider this when interpreting the results of the study. It is necessary for future studies to take this issue into account in order to strengthen the validity of these findings. Considering the sensitivity analysis and the low robustness to the assumption of pre-treatment confounding, it is necessary to reflect on the results with this in mind. In particular, genetic factors [55–57] may be important, but further unobserved factors may also be pre-treatment factors that are connected to education, diabetes, and cognitive function such as e.g. the socioeconomic background in childhood [58, 59].

The multifaceted relationship between education and cognition has already been pointed out, and several lifestyle factors that are related to education, diabetes and cognition have been controlled in the model. Nevertheless, the complex relationship with nutrition could not be modelled, even if obesity was included in the statistical model. However, we did not find a significant pathway via hypertension, high cholesterol, or obesity in our statistical model which strengthens the importance of diabetes as one of the multifaceted pathways between education and cognition.

Further issues concern the analyzed sample. Population-based health surveys are typically affected by selection or response bias, which leads to a healthier study sample than in the underlying population. This is also suspected here, with a diabetes prevalence of 5.7% for the study sample (age 50+) towards 7.5% in the Netherland population aged 20–79 [60]. It is reasonable to assume that this also applies to individuals with impaired cognitive function. This may result in an underestimation of the effects in the statistical models and with it the size/proportion of the indirect effect of education on cognitive function. Moreover, the follow-up period between the baseline and second assessment was relatively short given the slow progression of diabetes and cognitive decline.

The relationship between education and cognitive abilities is well established and again evidenced by the findings of this study. Education, particularly in older age, is not a modifiable risk factor, whereas diabetes is. Thus, in the multifaceted relationship between education and cognition, diabetes represents one promising approach to modifying or preventing the risk of cognitive decline thereby counteracting the disadvantages of less education. This holds true, even if the association between education and cognitive function may be mediated by a series of factors, among them most prominently cognitive reserve.

Further research is needed to reveal how cognitive performance changes over time in the different educational groups. Additionally, the interplay between genetic predisposition, education and cognitive function should be explored.

## Conclusion

This study found that people with lower levels of education were more likely to have diabetes and that diabetes was associated with poor cognitive function. Most importantly, this study is the first to demonstrate that a part of the effect of education on cognitive function runs through diabetes. While the relationship between education and cognition is multifaceted, these findings emphasize the potentially importance of diabetes in explaining the role of education in promoting healthy cognitive function and mitigating the risk of cognitive decline. Lower and middle educated people are double disadvantaged with respect to cognitive function through a higher risk for diabetes as well as a lower cognitive reserve resulting from lower education. Early detection and treatment of diabetes may be particularly beneficial for these individuals to maintain good levels of cognitive function.

## Electronic supplementary material

Below is the link to the electronic supplementary material.

Supplementary Material 1

## Data Availability

Data may be obtained from a third party and is not publicly available. Researchers may apply to use the Lifelines data used in this study. For information on how to request Lifelines data and terms of use are available on their website at (https://www.lifelines.nl/researcher/how-to-apply).

## Abbreviations

ACME: Average causal mediation effect
CI: Confidence interval
HbA1c: Glycated hemoglobin (A1c)
mmol/L: Millimole per liter
SD: Standard deviation
UMCG: University Medical Center Groningen
