# Supplementary material for: The effect of diabetes in the multifaceted relationship between education and cognitive function
Source: BMC Public Health. 2024 Sep 27;24:2584. doi: 10.1186/s12889-024-20156-x (PMC11429487; doi:10.1186/s12889-024-20156-x)
Supplement: Supplementary file 1 — Supplementary Material 1 [file 12889_2024_20156_MOESM1_ESM.docx]

**The effect of diabetes in the multifaceted relationship between education and cognitive function – Supplemental material**

Supplemental Figures

[Supplementary Figure S1 2](#_Toc175831604)

[Supplementary Figure S2 3](#_Toc175831605)

Supplemental Tables

[Supplementary Table S1 4](#_Toc175831606)

[Supplementary Table S2 4](#_Toc175831607)

[Supplementary Table S3 5](#_Toc175831608)

[Supplementary Table S4 6](#_Toc175831609)

[Supplementary Table S5 7](#_Toc175831610)

# Supplementary Figure S1


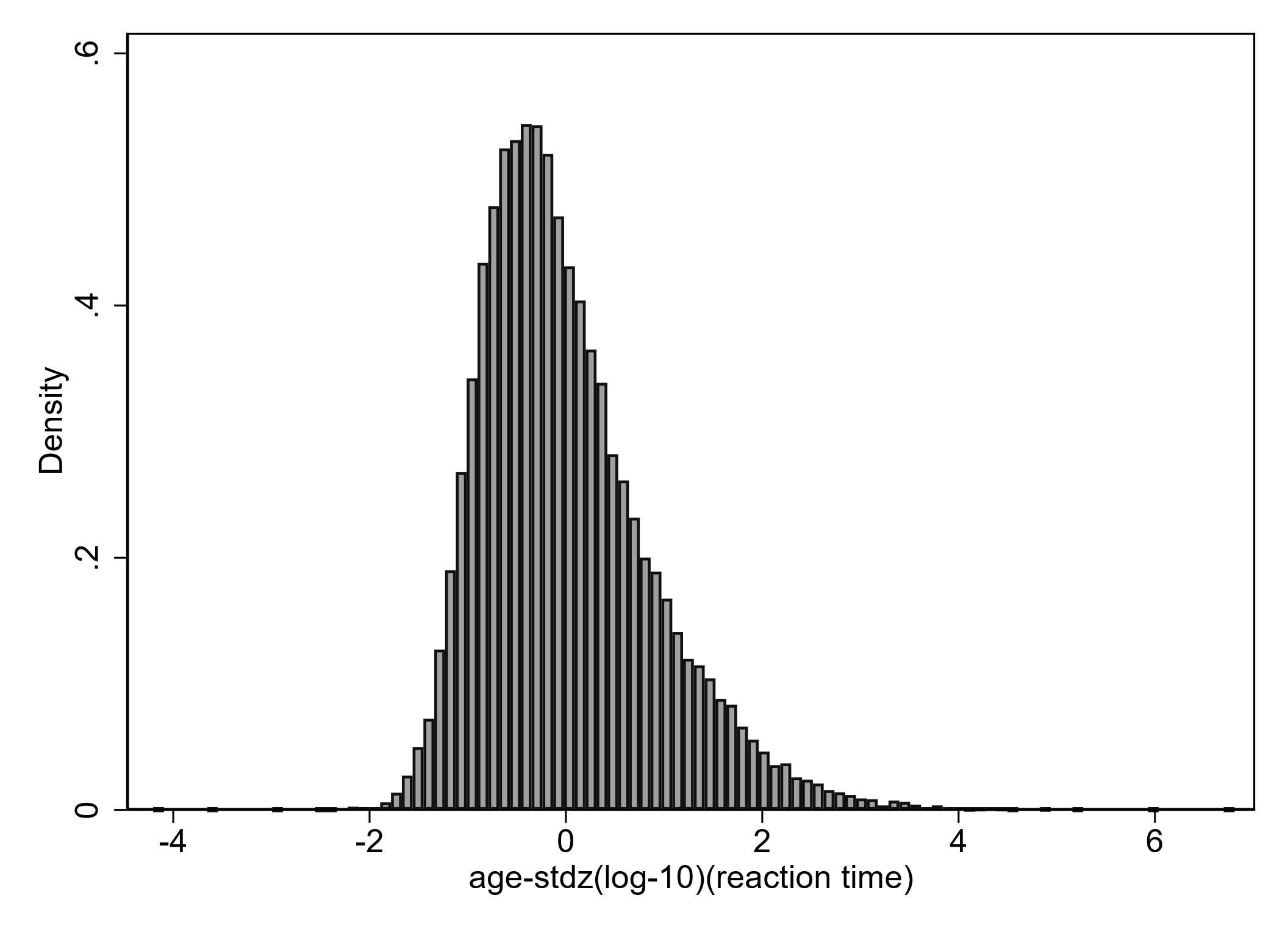


Figure S 1: Distribution of the combined outcome measure, source: lifelines data 2006-2015, own calculation

# Supplementary Figure S2


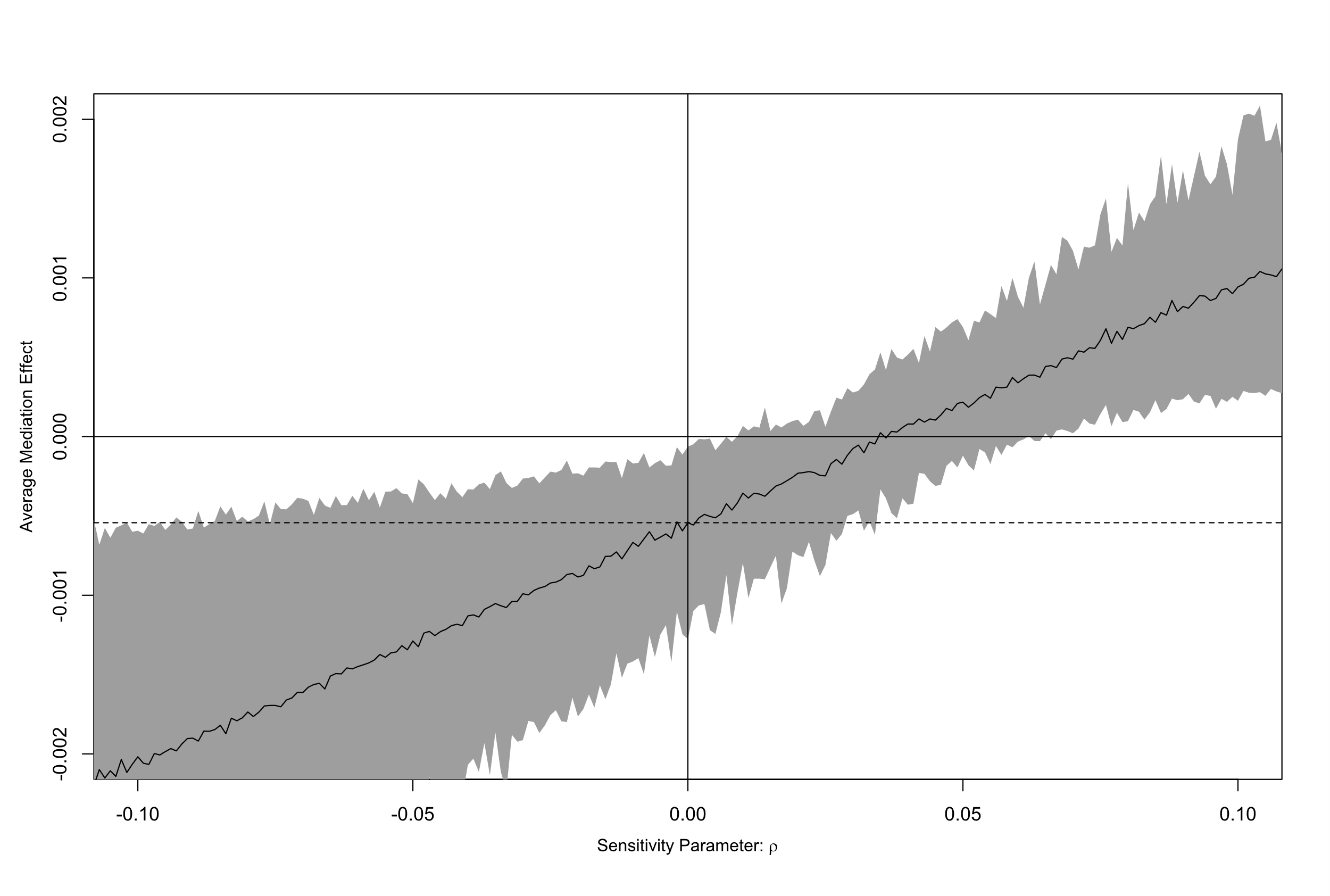


Figure S 2: Sensitivity analysis for the sequential ignorability assumption for the causal mediation approach, source: lifelines data 2006-2015, own calculation

# Supplementary Table S1

|  | | **Mediator model *** | | **Outcome model**** | | |
| --- | --- | --- | --- | --- | --- | --- |
| *dependent variable:* | | HbA1c-Value | | cognitive function | | |
| *model type:* | | OLS | | OLS | | |
|  | | **Reg. coef. (95% CI)** | | **Reg coef. (95% CI)** | | |
| **High education**  (Ref.: low-middle) | | -0.019753  ( -0.0313735; -0.0081325) | | -0.2021998  (-0.2245576; -0.1798419) | | |
| **HbA1c-value** | |  | | 0.0294949  (0.0057872; 0.0532027) | | |
| **Decomposition into direct, indirect and total effect** | | | | | | |
|  | **Direct effect (95% CI)** | | **Indirect effect (95% CI)** | | **Total effect (95% CI)** |  |
| **High education** | -0.2021998  (-0.2245576; -0.179842) | | -0.0005826  (-0.001148; -0.0000172) | | -0.2027824  (-0.225144; -0.180421) |  |

Table S 1: Sensitivity analysis. structural equation model, regression coefficients and 95% confidents intervals and decomposition into direct, indirect, and total effect of education, source: lifelines data 2006-2015, own calculation
* Model controlled for: Age, sex, physical activity, obesity, smoking history, income, and hypertension
** Model controlled for: age, sex, physical activity, obesity, smoking history, income, comorbidities, and cognition test accuracy

# Supplementary Table S2

|  | **model 1 ***  **(mediator)** | **model 2 ****  **(outcome)** |
| --- | --- | --- |
| *dependent variable:* | hypertension | cognitive function |
| *model type:* | logistic | OLS |
|  | **Reg. coef. (95% CI)** | **Reg coef. (95% CI)** |
| **High education**  (Ref.: low-middle) | -0.0546924  (-0.116416; 0.0070313) | -0.2022816  (-0.2246472; -0.1799161) |
| **Hypertension**  (Ref.: No-hypertension) |  | 0.0160565  (-0.0067055; 0.0388185 ) |
| **(Pseudo) R-squared** | 0.0342 | 0.0847 |
| **Number of observations** | 26,131 | 26,131 |
| **ACME of education (indirect effect)** | -0.0002734 (-0.0008386; 0.0001067 ) | |
| **direct effect of education** | -0.2024666 (-0.2251612; -0.180523) | |

Table S 2: Results of regression models for the mediator and the outcome variable, regression coefficients and 95% confidents intervals and direct & indirect effect of education from the causal mediation analysis, source: lifelines data 2006-2015, own calculation.
* Model controlled for: Age, sex, physical activity, obesity, smoking history, and income.
** Model controlled for: age, sex, physical activity, obesity, smoking history, income, diabetes, comorbidities, and cognition test accuracy

# Supplementary Table S3

|  | **model 1 ***  **(mediator)** | **model 2 ****  **(outcome)** |
| --- | --- | --- |
| *dependent variable:* | high cholesterol | cognitive function |
| *model type:* | logistic | OLS |
|  | **Reg. coef. (95% CI)** | **Reg coef. (95% CI)** |
| **High education**  (Ref.: low-middle) | -0.202095  (-0.2723973; -0.1317927 ) | -.2022816  (-0.2246472; -0.1799161) |
| **High cholesterol**  (Ref.: Not-High cholest.) |  | 0.0155597  (-0.0098537; 0.0409731) |
| **(Pseudo) R-squared** | 0.0640 | 0.0847 |
| **Number of observations** | 26,131 | 26,131 |
| **ACME of education (indirect effect)** | -0.0005781 (-0.0016567; 0.0003778) | |
| **direct effect of education** | -0.2024666 (-0.2251612; -0.180523) | |

Table S 3: Results of regression models for the mediator and the outcome variable, regression coefficients and 95% confidents intervals and direct & indirect effect of education from the causal mediation analysis, source: lifelines data 2006-2015, own calculation.
* Model controlled for: Age, sex, physical activity, obesity, smoking history, income, and hypertension
** Model controlled for: age, sex, physical activity, obesity, smoking history, income, comorbidities (excl. high cholesterol), and cognition test accuracy

# Supplementary Table S4

|  | **model 1 ***  **(mediator)** | **model 2 ****  **(outcome)** |
| --- | --- | --- |
| *dependent variable:* | obesity | cognitive function |
| *model type:* | logistic | OLS |
|  | **Reg. coef. (95% CI)** | **Reg coef. (95% CI)** |
| **High education**  (Ref.: low-middle) | -0.3257572  (-0.4098978; -0.2416165) | -0.2022816  (-0.2246472; -0.1799161 ) |
| **obesity**  (Ref.: No-obesity) |  | -0.0023241  (-0.0311638; 0.0265156) |
| **(Pseudo) R-squared** | 0.0573 | 0.0847 |
| **Number of observations** | 26,131 | 26,131 |
| **ACME of education (indirect effect)** | 0.0001187 (-0.0012241; 0.0014437) | |
| **direct effect of education** | -0.2024666 (-0.2251612; -0.180523) | |

Table S 4: Results of regression models for the mediator and the outcome variable, regression coefficients and 95% confidents intervals and direct & indirect effect of education from the causal mediation analysis, source: lifelines data 2006-2015, own calculation.
* Model controlled for: Age, sex, physical activity, obesity, smoking history, income, and hypertension
** Model controlled for: age, sex, physical activity, smoking history, income, diabetes, comorbidities, and cognition test accuracy

# Supplementary Table S5

|  | **model 1 ***  **(mediator)** | **model 2** †  **(outcome)** |
| --- | --- | --- |
| *dependent variable:* | diabetes status | cognitive function |
| *model type:* | logistic | OLS |
|  | **Reg. coef. (95% CI)** | **Reg coef. (95% CI)** |
| **High education**  (Ref.: low-middle) | -0.1551 (-0.2728; -0.0375) | -0.2022 (-0.2246; -0.1798) |
| **Diabetes**  **baseline & follow-up**  (Ref.: No-Diabetes) | - | 0.0720 ( 0.0324; 0.1116) |
| **(Pseudo) R-squared** | 0.1062 | 0.0849 |
| **Number of observations** | 26131 | 26131 |
| **ACME of education (indirect effect)** | -0.00090 (-0.00184; -0.00026)  0.44% of the total effect | |
| **direct effect of education** | -0.20239 (-0.22508; -0.18045) 99,56% of the total effect | |
| **total effect of education** | -0.20330 (-0.22654; -0.18090) | |

*Table S 6: Results of regression models for the mediator and the outcome variable.
Regression coefficients and 95% confidents intervals & direct, indirect and total effect of education from the causal mediation analysis, source: lifelines data 2006-2015, own calculation.*** Model controlled for: Age, sex, physical activity, obesity, smoking history, income, and hypertension
† Model controlled for: age, sex, physical activity, obesity, smoking history, income, comorbidities, and cognition test accuracy*
